# Supplementary material for: Adaptation of a Commercial NAD+ Quantification Kit to Assay the Base-Exchange Activity and Substrate Preferences of SARM1
Source: Molecules. 2024 Feb 14;29(4):847. doi: 10.3390/molecules29040847 (PMC10891823; doi:10.3390/molecules29040847)
Supplement: Supplementary file 1 [file molecules-29-00847-s001.zip › molecules-2842287-supplementary.pdf]

SUPPLEMENTAL INFORMATION

(associated to Figure 2 & Figure 3)

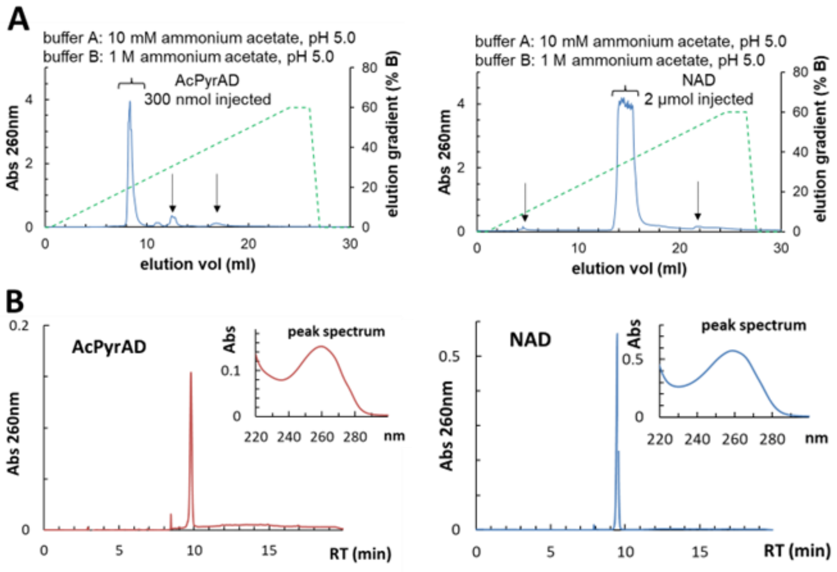

Figure S1. IEC-FPLC purification of commercial stocks of either NAD or AcPyrAD

(**A**) Typical chromatographies of AcPyrAD (left) or NAD (right) carried out for cleaning. Purchased dinucleotides were dissolved in water and injected on TSK-DEAE (Tosoh column, 250x4.6mm) equilibrated in buffer A, followed by elution at 1 ml/min by a gradient up to 60% of buffer B as indicated. Peaks below parentheses were collected and lyophilized. Black arrows indicate the most frequent contaminants removed. (**B**) C18-HPLC analysis of the two purified compounds resuspended in milliQ water. Insets, UV scan profiles of the single peaks eluted.

(associated to Figure 4)

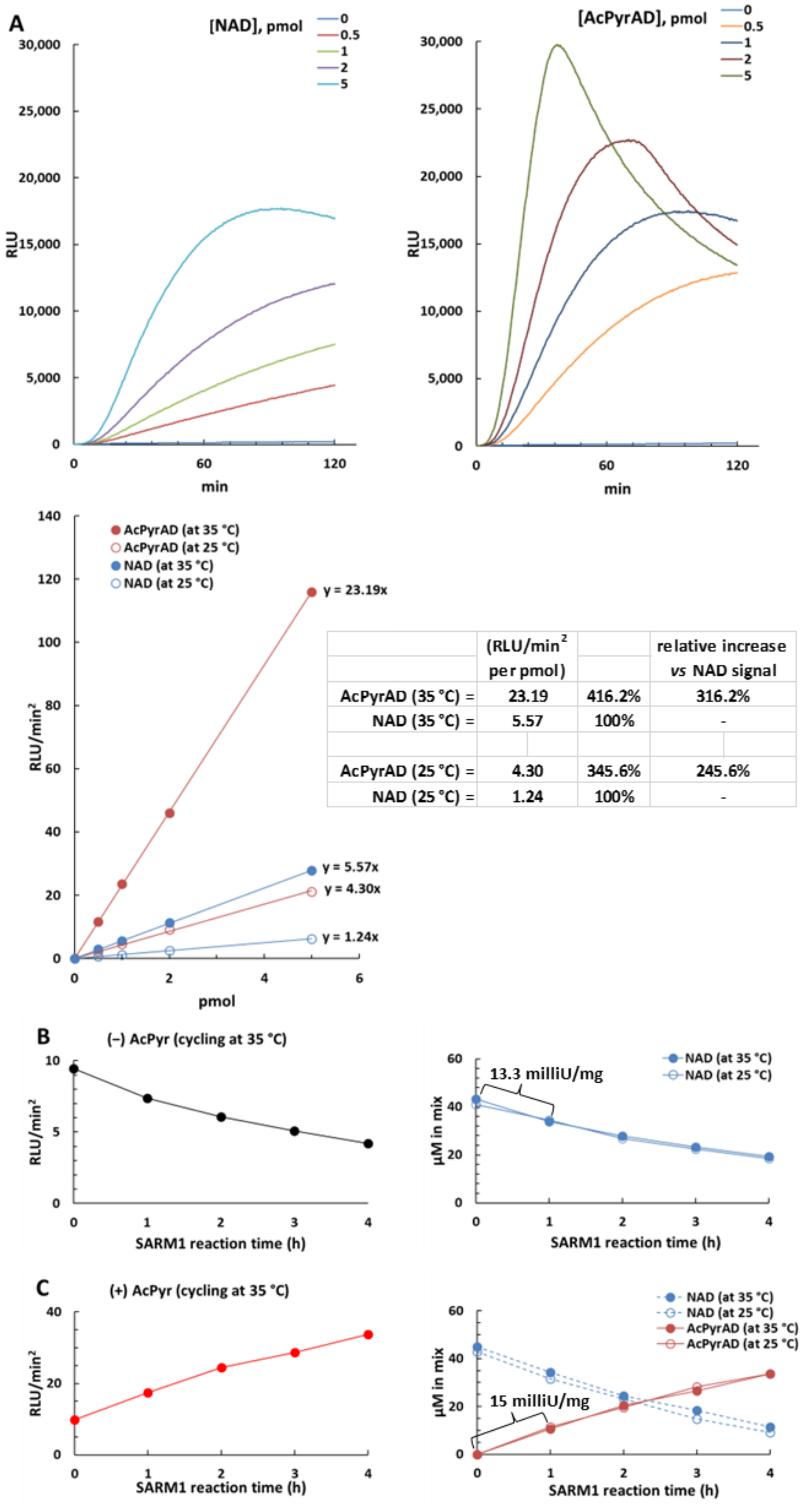

Figure S2. Cycling amplification of NAD or AcPyrAD at 35 °C & re-building at this higher temperature of data in Figure 4. (**A**) Luminescence emission (RLU) from 0 to 5 pmol of each NAD and AcPyrAD after cycling at 35 °C, followed by RLU/min² calculation and plotting as above. The

cycling slopes obtained at 35 °C for these two standards resulted higher than previous values at 25 °C (see table data and both Fig 2A & Fig 3A of main text). Nonetheless, the relative increase of luminescence from AcPyrAD referred to that from NAD at each temperature is largely equivalent. Linearization showed R^2 values ≥ 0.99 in all cases. (**B**,**C**) SARM1 reactions (already analyzed, see Fig 4, main text) were re-evaluated through the NAD/NADH-Glo™ Assay at 35 °C. Data are color-coded like in Figure 4A. The results are superimposable to those obtained previously at 25 °C (open circles).

(associated to Figure 4)

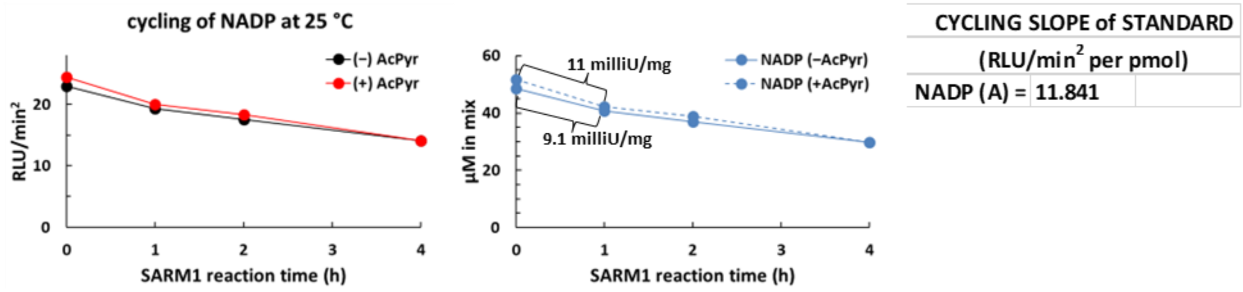

Figure S3. No AcPyr base exchange on NADP by SARM1 is measured by the NADP/NADPH-Glo™ Assay. Reaction mixtures contained recombinant SARM1 and 50 µM NADP as substrate, plus or minus 2 mM AcPyr. They were treated and processed as the mixtures with NAD (see Fig 4, main text) but cycled through the NADP/NADPH-Glo™ Assay at 25 °C together with a NADP standard. Luminescence emitted (RLU/min^2 values) was used to calculate the levels of NADP in the control minus AcPyr (continuous blue line) or in the sample plus AcPyr (dotted blue line). Calculations were made using equation 1 in Methods and the standard slope A for NADP as indicated. NADP consumption rates were calculated within the first one hour of incubation.

(associated to Figure 4)

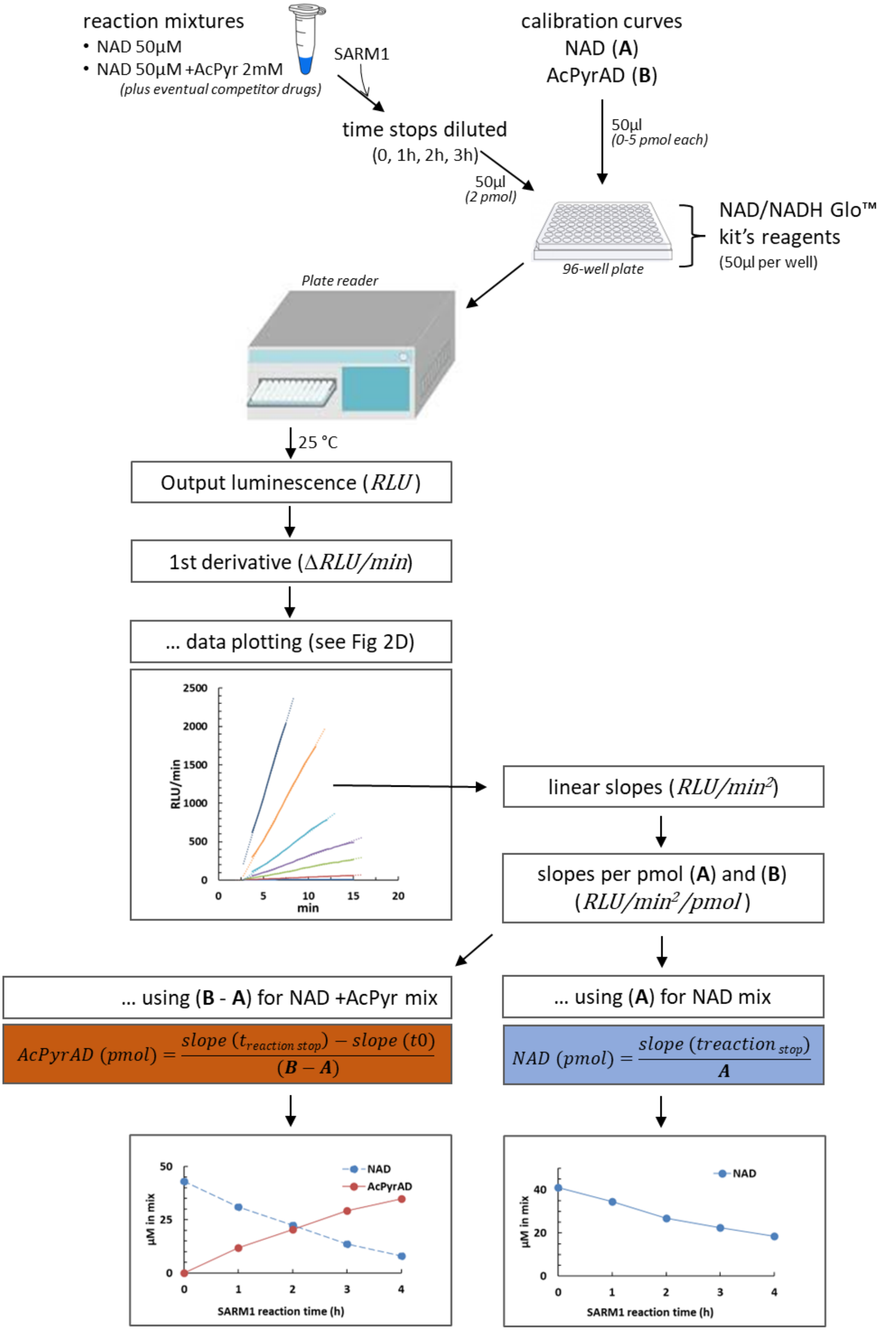

Figure S4. Schematic workflow of our proposed method to assay AcPyr base exchange activity of SARM1. The scheme depicts the assay described in Methods and in main text (Figure 4A).
